# Decreasing Costs of Dissemination of Research Results by Publishing in Diamond Open Access Journals

**Published:** 2022-03

**Authors:** Vladimir Mrša

**Affiliations:** Editor-in-Chief of Food Technology and Biotechnology President of Croatian; Association for Scholarly Communication; University of Zagreb, Faculty of Food Technology and Biotechnology,; Pierottijeva 6, 10 000 Zagreb, Croatia

It is our pleasure to announce the 60^th^ volume of *Food Technology and Biotechnology*, celebrating 60 years of bringing new scientific discoveries over to our readers, serving as a communication channel for scientists in the fields of food technology, biotechnology, and nutrition all over the world. In these 60 years we have grown from a local institutional newsletter to a globally present scientific journal. It has been an amazing journey in which the high quality research presented in a reader-friendly up-to-date fashion has always been our priority. The journey is not over and we are looking forward to our next 60 years.

In the first issue of volume 60 you can read papers covering different topics from microbial biotechnological production using *Providencia stuartii,* or *Saccharomyces cerevisiae*, the power of mushrooms *Coriolus versicolor,* or *Lignosus rhinocerus,* or the production of wine and mead. Papers also deal with the antioxidants in food, novel technologies in food processing, and the functional properties of microalgae in Ricotta.

As always, you can read these articles for free, with neither you nor your institution having to pay for their access. The authors did not have to pay for publishing their manuscripts either. *Food Technology and Biotechnology* is a so-called *diamond open access journal.* It means that its budget is provided by financial supports of public institutions like the Croatian Ministry of Science and Education, Croatian Academy of Science and Arts, Croatian Society for Biotechnology, as well as the publisher – Faculty of Food Technology and Biotechnology of the University of Zagreb. Diamond open access journals constitute a rather small share of scientific journals in science communication spectrum in which the financiers are neither readers (through institutional library subscriptions), nor authors through article processing charges. Although the number of papers published in diamond OA journals is not high, they are often referred to as the publishing model of the future. The financial pattern in which journals are financed by public institutions, ministries or other state bodies like universities or professional associations avoids high charges imposed by private publishers, liberating more funds for direct research costs, or scientific infrastructure. The model is in line with the ultimate intentions announced by the cOAlition S and formulated in Plan S (*1*), although other business models for scientific publishing are discussed within this plan, as well. At first sight, diamond OA journals seem like the best solution both for the researchers aiming to publish their results without devoting much of their project funds for this purpose, and to those aiming to access them freely and easily. However, public financing may have pitfalls of their own. Stable long-term financing may be a problem for smaller professional associations whose income may vary significantly from year to year and may depend on the current leadership. Such societies may lose motivation to maintain a journal, particularly if it does not gain any income but whose publishing creates a significant expense. Universities and larger societies with higher annual income may prove as more stable financiers as scientific communication is a part of their ’core business’. Indeed, considering technical possibilities and informatics infrastructure in place at most universities, scientific publishing should not present a significant financial burden. Actually, most diamond access journals are indeed funded by universities (*2*). On the other hand, journals financed by state public institutions like ministries, public foundations or other bodies distributing public funds may depend on the current political option and their changes may lead to different political decisions reflecting on science budgets and, consequently, scientific journal financing. Besides, it should be noted that some of the high budget professional associations create most of their incomes through publishing activities, sometimes engaging large publishers for their journals. For these societies a turn towards diamond open access would require a significant change in the structure of their annual income. Thus, in a system in which a larger segment of scientific results would be published in diamond open access journals, finding stable sources of income would be a difficult but indispensable task for scientific journal publishers. This conclusion has been strongly corroborated by a large study funded by Science Europe in order to gain a better insight in the OA diamond landscape (*2*). The study estimated the number of diamond open access journals at around 29 000. Most of these journals are not included in DOAJ, they are smaller in size and publish less than 25 papers per year, many of them are issued annually, and most of them belong to social sciences and humanities. The majority of them are published in Europe and South America by small publishers who publish between 1 and 5 journals. More than 70% of diamond OA journals are published by universities, around 15% by publishing companies, while 10% belong to professional associations. Concerning their operation and financing, most diamond open access journals face operational challenges and rely heavily on the efforts of volunteers. As such, they declare a need to develop infrastructure and to increase funding to support their operations. Securing sufficient and stable funding from sources who would not gain profit from publishing may at least partly be facilitated by decreasing the costs and the overall budget of the journal. More than 70% of diamond OA journals have an annual budget lower than 10 000 euro. This, however, contradicts the increasing demands of the scientific community for fast, simple, and high-quality publishing process. A variety of informatics tools designed for handling manuscripts, correspondence among authors, editors and reviewers, as well as on-line publishing with concomitant abandoning printed versions may lead to less expensive dissemination of scientific results. Development of such tools and their distribution among journals, as well as their proper use with the aim of maintaining high quality requires joint activities within the diamond OA journal community and incorporation of research institutions who have expertise required for the development of these tools. In this way, the scientific community itself could do something to improve the publication process and make it less expensive and dependent on large publishing companies. This should, in turn, be recognized by public science financiers, as well, since ensuring sufficient funding of projects directed to increasing the technical capacities for research dissemination could improve operation of diamond open access journals and provide financial benefits for the scientific community in long term.
